# Association Between Heme Oxygenase-1 Promoter Polymorphisms and the Development of Albuminuria in Type 2 Diabetes: A Case–Control Study

**DOI:** 10.1097/MD.0000000000001825

**Published:** 2015-10-30

**Authors:** Eun Young Lee, Yong-ho Lee, Soo Hyun Kim, Kyu Sik Chung, Obin Kwon, Beom Seok Kim, Chung Mo Nam, Chun Sik Park, Byung-Wan Lee, Eun Seok Kang, Bong-Soo Cha, Hyun Chul Lee

**Affiliations:** From the Department of Internal Medicine, College of Medicine, Catholic University of Korea, Seoul, Korea and Division of Endocrinology and Metabolism, Department of Internal Medicine, Seoul St. Mary's Hospital, Catholic University of Korea, Seoul, Korea (EYL); Department of Internal Medicine, Graduate School, Yonsei University College of Medicine, Seodaemun-gu, Seoul, Republic of Korea (EYL); Division of Endocrinology and Metabolism, Department of Internal Medicine, Yonsei University College of Medicine, Seodaemun-gu, Seoul, Republic of Korea (Y-HL, OK, B-WL, ESK, B-SC, HCL); Institute of Endocrine Research, Yonsei University College of Medicine, Seoul, Republic of Korea (Y-HL, SHK, OK, B-WL, ESK, B-SC, HCL); Division of Gastroenterology, Department of Internal Medicine, Yonsei University College of Medicine, Seodaemun-gu, Seoul, Republic of Korea (KSC); Division of Nephrology, Department of Internal Medicine, Yonsei University College of Medicine, Seodaemun-gu, Seoul, Republic of Korea (BSK); Department of Preventive Medicine, Yonsei University College of Medicine, Seodaemun-gu, Seoul, Republic of Korea (CMN); and Department of Physiology, Asan Medical Center, University of Ulsan College of Medicine, Seoul, Republic of Korea (CSP).

## Abstract

Heme oxygenase (HO)-1 is a key enzyme in cytoprotective mechanisms against oxidative stress in the cardiovascular-renal system. The T(-413)A single nucleotide polymorphism (SNP) and (GT)_n_ microsatellite polymorphism in the HO-1 gene promoter modulate the HO-1 gene transcriptional activity and these polymorphisms are associated with various human diseases.

We investigated the association between HO-1 promoter polymorphisms and nephropathy in type 2 diabetes. We sequenced the T(-413)A SNP and (GT)_n_ repeat segments of the HO-1 gene promoter in 536 patients with type 2 diabetes. (GT)_n_ alleles were divided into 2 groups: short (S, ≤25 GT repeats) and long (L, >25 GT repeats) alleles. The presence of albuminuria was used as a marker of diabetic nephropathy.

Patients with the TT genotype in the T(-413)A SNP were significantly more susceptible to albuminuria development than those carrying the A allele, with an odds ratio of 1.577 (95% confidence interval, 1.088 − 2.285; P = 0.016). Subgroup analysis showed that patients carrying the TT genotype with long duration of diabetes (≥20 years), poor glycemic control, male gender and without hypertension had higher odds ratios for the development of albuminuria. In vitro, promoter activity of the T(-413)A SNP was higher with A allele than T allele. Regarding to the (GT)_n_ repeats, the LL genotype showed a higher odds ratio for the development of albuminuria only in patients with hypertension when compared to the S allele.

In conclusion, the T(-413)A SNP in the HO-1 promoter is significantly associated with albuminuria development in type 2 diabetes patients, especially with longer duration and poor glycemic control.

## INTRODUCTION

Diabetic nephropathy is a major cause of diabetes-related morbidity and mortality, and its worldwide incidence has significantly increased for decades.^1–3^ Several cross-sectional and prospective studies have identified hypertension, hyperglycemia, dyslipidemia, male, race, and genetic susceptibility as important risk factors of diabetic nephropathy.^4^ Although hyperglycemia is an important factor causing diabetic complications, only one-half of diabetic patients with poor glycemic control develop diabetic nephropathy. In addition, some patients develop diabetic nephropathy in spite of good glycemic control.^5^ These suggest that unidentified genetic determinants increasing susceptibility to diabetic nephropathy may exist. Recent studies have demonstrated that polymorphisms in several candidate genes, such as the angiotensin 1-converting enzyme and carnosine genes, are likely associated with increased susceptibility,^6,7^ further implying the importance of genetic factors.

Increasing evidence indicates that excess oxidative stress caused by chronic hyperglycemia may play a pivotal role in pathogenesis of diabetic nephropathy. Superfluous reactive oxygen species (ROS) can stimulate the expression of cytokines leading to damage of podocytes and mesangial cells, which are crucial for maintenance of glomerular function.^8^ Heme oxygenase (HO)-1 is a key enzyme in cytoprotective mechanisms against oxidative stress in the cardiovascular-renal system.^9^ HO-1 is an inducible isoform of HO and is a rate-limiting enzyme in heme degradation leading to the generation of free iron, carbon monoxide, and biliverdin, which is subsequently converted into bilirubin. These substances produced by HO-1 have anti-inflammatory and anti-oxidative effects, and these effects are compatible with the findings observed in animal models of diabetic kidney injury that the physiologic overexpression of HO-1 functions to protect renal cells from oxidative stress.^9,10^

Recently, several studies suggested that polymorphisms in the promoter region of the HO-1 gene might modulate the transcriptional level of HO-1 and have significant associations with human diseases related to the vascular system.^11^ Three promoter polymorphisms of the HO-1 gene have been identified: one (GT)_n_ microsatellite length polymorphism and 2 single nucleotide polymorphisms (SNPs), G(-1135)A and T(-413)A.^12^ Long (GT)_n_ repeats are linked to lower transcriptional activity,^13^ higher prevalence of coronary artery disease in patients with diabetes,^14^ vulnerability to kidney injury, and dysfunction after renal transplantation.^15^ The AA genotype of the T(-413)A SNP leads to a higher expression of HO-1 and is associated with a lower incidence of ischemic heart disease in a Japanese population.^16^ However, the influence of HO-1 genotypes in diabetic nephropathy has not yet been assessed. Considering the clinical relevance of HO-1 gene promoter polymorphisms to vascular complications, seeking a similar relevance of these types of polymorphisms to diabetic nephropathy development seemed worthwhile. Thus, we investigated the role of polymorphisms in the promoter region of HO-1 as a predictive biomarker for development and prognosis of albuminuria, as an index of diabetic nephropathy.

## PATIENTS AND METHODS

### Study Subjects

A total of 536 Korean type 2 diabetic patients were recruited from the Diabetes Center in Severance Hospital. According to the criteria of the American Diabetes Association,^17^ diabetes was diagnosed with any 1 of the following criteria: fasting blood glucose level of 126 mg/dL or higher; random blood glucose level of 200 mg/dL or higher and symptoms of diabetes such as increased thirst, urination, or weight loss; blood glucose level of 200 mg/dL or higher after 2 hr of 75 g oral glucose tolerance test; and glycated hemoglobin (HbA1c) level of 6.5% or higher. Patients were excluded if they were using glucocorticoid medication or had type 1 diabetes mellitus, abnormal thyroid stimulating hormone levels, a severe infectious disease or a history of cancer, kidney transplantation, ketoacidosis, impending or undergoing dialysis, or liver disease. This research was carried out according to The Code of Ethics of the World Medical Association (Declaration of Helsinki). All patients received adequate information about the study and provided written informed consent. The protocol of this study was approved by the ethics committee of the Yonsei University College of Medicine.

### Clinical and Biochemical Measurements

A detailed medical history, including medications, and duration of diabetes, was recorded. All anthropometric and laboratory parameters including fasting plasma glucose (FPG), insulin, and lipid profiles were measured after 8 hr of fasting. HbA1c was measured 4 times over a 12- to 18-month period using high-performance liquid chromatography (Variant II; Greencross, Seoul, Korea) and mean HbA1c was used to represent overall glycemic control. The homeostasis model assessment (HOMA) of insulin resistance (IR) and HOMA-β were calculated by using the following formula: HOMA-IR = (insulin [μIU/mL] × FPG [mg/dL])/405, HOMA-β = 360 × insulin (μIU/mL)/(FPG [mg/dL] − 63). Hypertension was defined by a blood pressure ≥ 140/90 mm Hg or the use of antihypertensive medications.

As glomerular filtration rate is usually normal or increased during the early stage of diabetic nephropathy, albuminuria is considered as a marker for development of nephropathy in type 2 diabetes.^18^ Therefore, we used the presence of albuminuria as an indicator for developing diabetic nephropathy in this study. A random or 24-hr urine specimen was collected from all patients and the concentrations of albumin and creatinine were analyzed. The urinary albumin and creatinine concentration were determined by a nephelometric assay with sensitivity to 2.3 mg/L (Beckman Image, Fullerton, CA) and a colorimetric assay (Roche Diagnostic, Indianapolis, IN), respectively. The albumin/creatinine ratio (ACR) in randomly collected urine samples was measured more than twice during the follow-up. Significant albuminuria was defined as follows: urinary ACR ≥ 30 mg/g or albumin excretion ≥ 30 mg/day in a 24 hr urine analysis.

### DNA Extraction and Analysis of Polymorphisms in the HO-1 Gene Promoter

Genomic DNA was isolated from peripheral blood lymphocytes. The genotyping of the HO-1 promoter polymorphism T(-413)A (rs2071746) was performed using the TaqMan fluorogenic 5′ nuclease assay (ABI, Foster City, CA) as previously described.^19^ Forty-eight duplicate samples and negative controls were included to ensure the accuracy of genotyping, and 100% of the duplicates replicated the original genotype. The rate of successful genotyping was 96.5% for rs2071746 and 98.2% for rs3761439.

The length of the (GT)_n_ microsatellite polymorphism in the HO-1 gene promoter was determined as previously described with slight modification.^20^ The (GT)_n_ repeat segment was amplified in a volume of 20 μL containing 50 ng of genomic DNA, 0.5 pmol of each primer, 1× polymerized chain reaction (PCR) buffer, 200 μM dNTP, 1.5 mM MgCl_2_, 1× Band Doctor, and 0.5 U *Taq* polymerase (Solgent, Daejeon, Korea). The reaction consisted of denaturation at 95°C for 2 min, followed by 32 cycles of 95°C for 20 sec, 53°C for 40 sec, and 72°C for 1 min, with a final extension at 72°C for 5 min using PTC-200 thermal cycler (Bio-Rad, Hercules, CA). The PCR products were analyzed by capillary on a MegaBACE500 (GE Healthcare, Piscataway, NJ) together with an allelic ladder after denaturation at 96°C for 5 min.

### Expression Study of HO-1

To explore the regulatory effects of the T(-413)A polymorphism in the promoter region, we constructed HO-1 promoter/luciferase fusion genes. The promoter region between −1876 and +75 was amplified by PCR with a sense primer, 5′-TCCACCTCCACCTTCCCTTAAAGTCGGC-3′ and an antisense primer, 5′-ACGCTCGAGAGGAGGCAGGCGTTGACT-3′, and sub-cloned into pGL3-Basic or pGL3-Enhancer DNA (Promega, Madison, WI), which does not contain any promoter sequence. Site-specific mutation was made using Pfu-X DNA polymerase (SPX16-R250, Solgent). Transfection was performed in human embryonic kidney cells (HEK293, ATCC CRL-1573) and murine mesangial cells (SV40 MES-13, ATCC CRL-1927) using lipofectamine 2000 (Invitrogen, Carlsbad, CA) according to the manufacturer's instructions. Assays were performed 48 hr after transfection using the Dual-Luciferase Reporter Assay System (Promega). Luciferase activity was read with a microplate luminometer (Centro XS3 LB960, Berthold Technologies, Bad Wildbad, Germany). Transfections were carried out in triplicate in independent experiments.

### Statistical Analyses

The genotype frequencies were tested for Hardy–Weinberg equilibrium using the χ^2^ test. Continuous variables were expressed as the mean ± standard deviation; variables not normally distributed were expressed as median and interquartile ranges. The Student *t* test or χ^2^ test were used to compare the continuous or categorical parameters of groups distinguished by different genotypes or the presence of albuminuria. Mann–Whitney *U* test was used to compare the variables not having a normal distribution. Multivariable logistic regression tests were performed to control confounding variables, and odds ratio (OR) and 95% confidence intervals (CIs) were calculated. Genetic analysis using haplotypes was performed to evaluate the combined effects of both the T(-413)A SNP and the (GT)_n_ repeat polymorphism in the HO-1 promoter on the prevalence of albuminuria. Based on previous studies,^13,21^ the distribution of lengths of (GT)_n_ repeats was categorized into 2 subclasses: the short (S) allele consisted of ≤25 repeats, and the long (L) allele consisted of >25 repeats. Association between haplotypes and the prevalence of albuminuria in type 2 diabetes patients was analyzed using Haploview version 4.2 (Massachusetts Institute of Technology, Cambridge, MA). A *P* value <0.05 was considered statistically significant. Statistical analyses except analysis of haplotype were performed using SPSS for Windows software (version 18.0; SPSS, Chicago, IL).

## RESULTS

### Clinical Characteristics of the Subjects and Association Between Genotype and Albuminuria

As shown in Table 1, the mean age, body mass index, and duration of diabetes were significantly higher in patients with albuminuria than in those without albuminuria. Patients with albuminuria also had higher levels of HbA1c, HOMA-IR, and triglycerides and an increased quantity of urinary albumin excretion. Compared with diabetic patients without albuminuria, patients with albuminuria had a higher prevalence of hypertension, carotid plaques, retinopathy, and neuropathy.

**TABLE 1:**
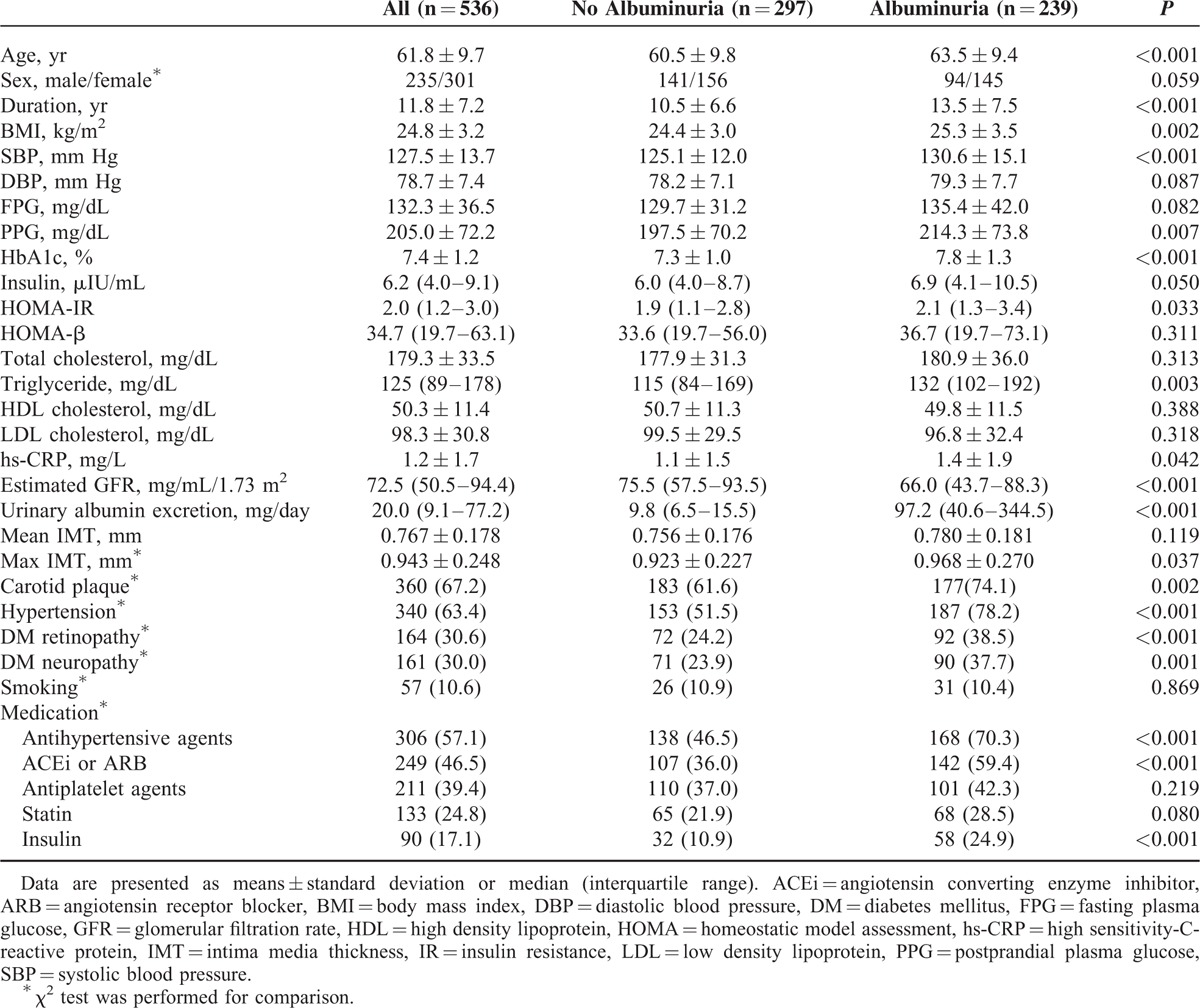
Clinical and Biochemical Characteristics of Study Subjects With and Without Albuminuria

Genotyping of the T(-413)A SNP in the HO-1 gene promoter was replicated for 297 patients without albuminuria and 239 patients with albuminuria. The distribution of the T(-413)A SNP was in agreement with Hardy–Weinberg equilibrium and the T was the major allele (53.4%). As shown in Table 2, the T(-413)A SNP was associated with albuminuria in additive and dominant models. Analysis of the (GT)_n_ microsatellite polymorphism was completed in only 427 patients due to lack of samples. A total of 25 different alleles for the HO-1 (GT)_n_ microsatellite were identified in patients with type 2 diabetes. The distribution of alleles for the (GT)_n_ microsatellite in HO-1 promoter ranged from (GT)_15_ to (GT)_40_. A bimodal pattern of (GT)_n_ alleles was observed with a peak at 23 (18.6%) and 30 (31.6%) repeats, which is consistent with other reports.^13,22^ Based on their distribution and previous studies,^13,21^ we divided the allelic repeats into 2 subclasses: short (S) allele with 25 or less (GT)_n_ repeats and long (L) allele with more than 25 (GT)_n_ repeats. The distribution of the (GT)_n_ polymorphism was in agreement with Hardy–Weinberg equilibrium and the L was the major allele (59.8%). The (GT)_n_ polymorphism was not associated with albuminuria development in additive, dominant, or recessive models (Table 2). The haplotype analysis of HO-1 promoter polymorphisms did not show any difference (data not shown).

**TABLE 2:**
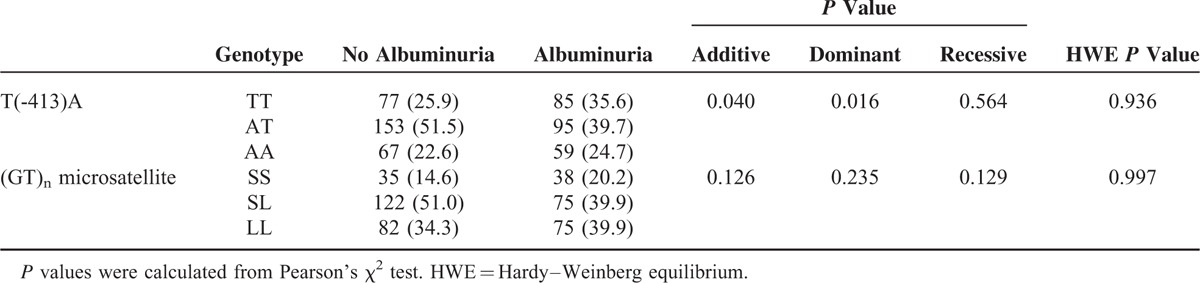
Genotype and Genetic Models Frequencies Between the Patients With and Without Albuminuria

### Clinical Characteristics of the Subjects According to the T(-413)A SNP

Based on the analysis of genetic models (Table 2), we compared the general characteristics in the patients according to the genotypes of the T(-413)A in the HO-1 promoter. Albuminuria was significantly more prevalent in patients with the TT genotype than in those with either the AA or AT genotype (52.5% vs 41.2%, *P* < 0.05). Fasting and postprandial glucose, HbA1c levels, and urinary albumin excretion were higher in patients with the TT genotype than in those with either the AA or AT genotype (each *P* < 0.05). No other clinical or biochemical parameters significantly differed between AA + AT and TT groups (Table 3).

**TABLE 3:**
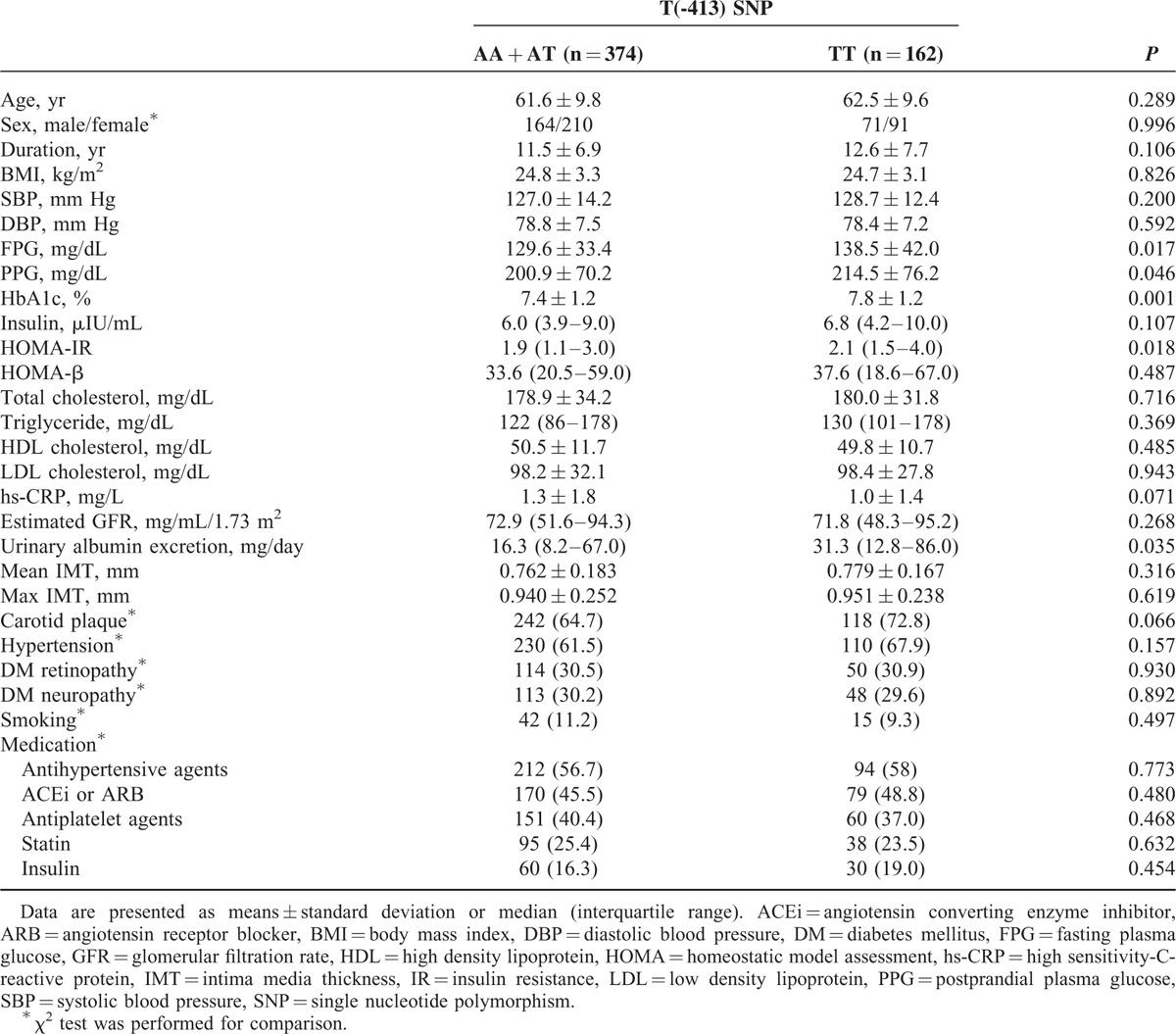
Clinical and Biochemical Characteristics of Study Subjects According to the T(-413)A SNP

### Analysis of the Contribution of HO-1 Promoter Polymorphisms to Albuminuria Development

**FIGURE 1:**
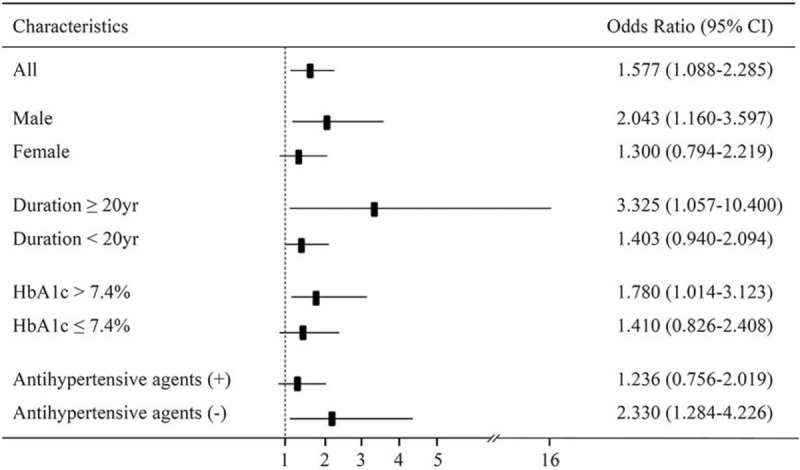
Odds ratio (OR with 95% confidence interval) of genotype (AA + AT/TT) for albuminuria in various subgroups of type 2 diabetic patients.

To evaluate the association of albuminuria with HO-1 genotypes or other conventional risk factors, multivariate logistic regression analysis was performed (Table 4). After adjusting for established variables, patients with the TT genotype were significantly more susceptible to albuminuria development compared with those carrying the A allele with an OR of 1.616 (95% CI, 1.007–2.593; *P* = 0.047). Other known risk factors, such as obesity, hypertension, and status of glycemic control, were also significantly associated with albuminuria, as expected. However, no significant relationship between carriers of the LL genotype and albuminuria was observed (data not shown). We then stratified the study population according to baseline clinical and biochemical characteristics and performed subgroup analyses to further investigate the association of the T(-413)A SNP with the risk of albuminuria in each subgroup (Fig. 1). After categorizing the patients based on the duration of diabetes, the OR of having albuminuria for patients with diabetes ≥20 years and with the TT genotype rose from 1.403 to 3.325 and reached statistical significance (95% CI, 1.057–10.400; *P* = 0.040). Furthermore, patients with poor glycemic control and male sex had higher ORs than those without these risk factors, and the ORs for patients with the TT genotype and these factors reached statistical significance. Although there was no significant association between (GT)_n_ repeats and albuminuria, patients with hypertension carrying the LL genotype had a higher OR of 1.825 (95% CI, 1.095–3.042; *P* = 0.021) than those carrying an SS or SL genotype.

**Table 4:**
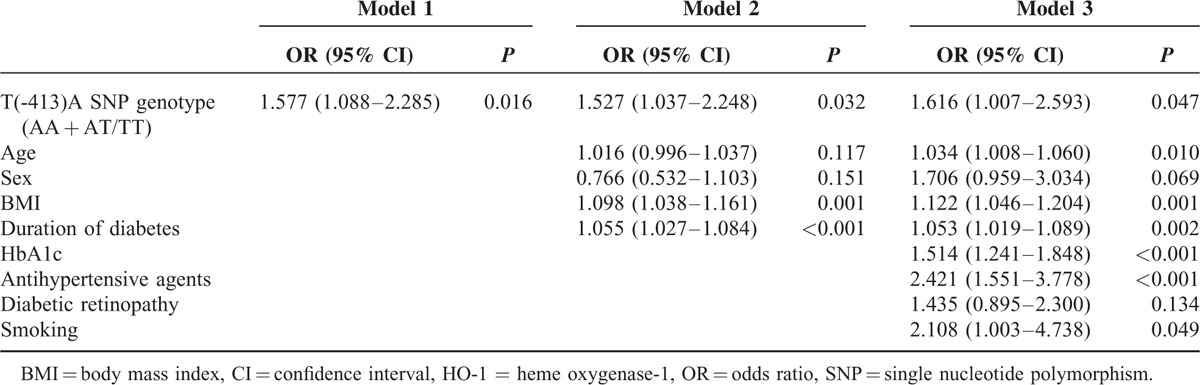
Logistic Regression Model Assessing the Independent Association of the HO-1 Genotype (AA + AT/TT) and Albuminuria in Diabetic Patients

### Functional Significance of HO-1 Promoter Polymorphisms

**FIGURE 2:**
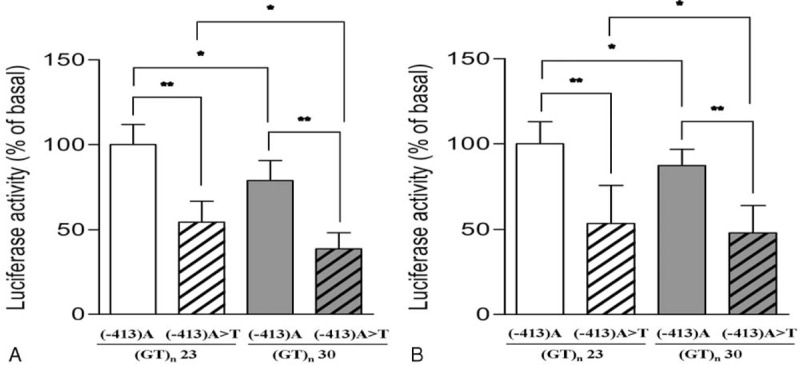
Assessment of HO-1 promoter activities. Transient transfection of HO-1 promoter/luciferase fusion genes was performed in HEK-293 cells (A) and mesangial cells (B). Luciferase activity, which indicated activity of the HO-1 promoter, was expressed as relative luciferase units. HO-1 = heme oxygenase-1. ∗*P* < 0.05, ∗∗*P* < 0.01.

To evaluate functional significance of HO-1 promoter polymorphisms, we compared levels of HO-1 expression according to the HO-1 promoter polymorphisms using an in vitro luciferase reporter assay. As shown in Figure 2, the in vitro promoter activity of the A allele of the T(-413)A SNP was significantly higher than that of the corresponding T allele (*P* < 0.01). Regardless of the T(-413)A SNP allele, the promoter activity of the (GT)_23_ allele was significantly higher than that of the (GT)_30_ allele (*P* < 0.05).

## DISCUSSION

In this retrospective analysis of diabetic patients, we found that certain polymorphisms of the HO-1 gene promoter are related to the development of albuminuria, which is 1 of the early findings of diabetic nephropathy. The A allele of the T(-413)A SNP was associated with a lower prevalence of albuminuria, while the TT genotype increased susceptibility to albuminuria. In addition, we demonstrated higher promoter activity with A allele of the T(-413)A SNP than T allele in vitro.

The Diabetes Control and Complications Trial and the United Kingdom Prospective Diabetes Study have clearly showed that long-term glycemic control in patients with diabetes is important for the prevention of microvascular complications, including nephropathy. Based on the role of hyperglycemia-induced ROS in the development of diabetic nephropathy, recently published studies have demonstrated in animal models that antioxidant therapy or induction of antioxidative enzymes effectively prevents the progression to nephropathy in diabetes.^23^ These findings suggest that inhibiting ROS generation by activation of HO-1 may be a promising approach to protect the kidneys from diabetic nephropathy. In terms of oxidative injury, HO-1 has been recognized as a key enzyme for attenuating the production of intracellular ROS by releasing antioxidative by-products from heme degradation. Induction of HO-1 expression is controlled by numerous transcription factors, including Nrf2 as well as noxious stimuli such as cytokines, endotoxins, oxidative stress, and hypoxia.^9,10^ Furthermore, several polymorphisms in the promoter of HO-1 gene also modulate its transcriptional activity. The functional importance of human HO-1 promoter polymorphisms has been intensively studied in various diseases and conditions,^11^ but no reports have suggested their association with the risk of diabetic nephropathy.

Unlike the (GT)_n_ microsatellite polymorphism, the T(-413)A SNP has not been investigated in detail with regard to its functional significance in human disease. Ono et al^16^ revealed that the AA genotype of the T(-413)A SNP was associated with a lower incidence of ischemic heart disease. In another study, graft survival after liver transplantation was significantly associated with the A allele, regardless of the (GT)_n_ microsatellite polymorphism,^24^ suggesting an organ-protective effect of the A allele, which is consistent with our findings. The interaction between gene and environmental or clinical factors is important in genetic epidemiology to understand the characteristics of gene polymorphisms. According to the subgroup analyses, patients with the TT genotype and prolonged duration of diabetes (≥20 years), poor glycemic control, or male gender were significantly more susceptible to albuminuria development than patients without these factors. This implies that genetic factors make patients more vulnerable to common aggravating conditions related to nephropathy. These patients may require stricter control of modifiable risk factors such as hyperglycemia to prevent renal complications. However, in terms of blood pressure, the TT genotype was associated with the risk of albuminuria in patients without hypertension. In our study, most diabetic patients with hypertension have taken an angiotensin converting enzyme inhibitor or angiotensin receptor blocker medication, which slows the progression of diabetic nephropathy and reduces albuminuria.^25,26^ This could interfere with genetic effects on the development of albuminuria in these patients.

Regarding the (GT)_n_ microsatellite polymorphism, we observed that patients with long (GT)_n_ repeats showed a lower level of HO-1 promoter activity compared to patients with short (GT)_n_ repeats. Considering the protective role of HO-1 on kidneys in animal studies,^27,28^ low expression of HO-1 by vulnerable genotypes of promoter polymorphisms may be associated with diabetic nephropathy development. However, our results showed that the (GT)_n_ microsatellite polymorphism had no significant association with albuminuria. In subgroup analysis, long (GT)_n_ repeats were associated with the risk of albuminuria development in patients with hypertension. With respect to the implication of HO-1 promoter polymorphisms in kidney disease, several studies have reported that short (GT)_n_ repeats were associated with a decreased incidence of chronic allograft nephropathy, resulting in better survival of grafted kidney.^15,29^ However, a subsequent study with more patients and longer follow-up duration revealed that there was no protective effect for the short (GT)_n_ repeats on graft survival.^30^ Studies of renal impairment due to IgA nephropathy, similar to the studies of renal transplantation, have shown conflicting results on the functional significance of short (GT)_n_ repeats.^31,32^ Additional study will be needed to determine whether (GT)_n_ microsatellite polymorphisms of HO-1 gene have functional significance in renal disease.

According to several studies on the expression of HO-1, including those using the luciferase assay, transcriptional activity of the HO-1 promoter is regulated by both the (GT)_n_ microsatellite polymorphism and the T(-413)A SNP. The HO-1 promoter activity of the A allele is significantly higher than that of the T allele regardless of the (GT)_n_ length in transfected bovine endothelial cells^16^ and human liver specimens.^24^ Other data demonstrated that short, rather than long, (GT)_n_ repeats correlated with increased HO-1 expression in human blood mononuclear cells^13^ and transfected rat smooth muscle cells.^33^ In our study, HO-1 promoter activities in the A allele and short (GT)_n_ repeat were significantly higher than that of the T allele and long (GT)_n_ repeat, respectively. To date, the molecular mechanisms by which the (GT)_n_ repeat and T(-413)A SNP modulate HO-1 promoter activity are unidentified. Numerous stress-responsive transcription factors bind to both the proximal promoter and distal 5′ enhancer regions and regulate HO-1 gene transcription.^12,34^ Therefore, these polymorphisms may induce conformational changes of adjacent regulatory elements to modulate the accessibility of proteins.^11^ Additionally, unidentified functional genetic variants that are located in distal regions of the HO-1 promoter may be in linkage disequilibrium with the T(-413)A SNP because there are consensus binding sites before −1 kb for important transcription factors, including Nrf2.^34^

The present study has limitations that may be overcome by further investigations. First, we did not measure the bilirubin or ferritin levels to analyze their association with these polymorphisms and diabetic nephropathy. Second, the number of patients in our sample was rather small and all were Asian; therefore, we could not detect variations that may exist among different races or ethnicities.

In conclusion, we demonstrated for the first time that the T(-413)A SNP in the HO-1 promoter region was associated with the prevalence of albuminuria, especially in patients with long duration of diabetes, poor glycemic control, and male gender. The (GT)_n_ microsatellite polymorphism in the HO-1 promoter showed association with albuminuria only in patients with hypertension. Further studies are necessary to elucidate the exact mechanism for modulating the HO-1 promoter and its clinical implications for diseases related to oxidative stress.
