# Association Between Heme Oxygenase-1 Promoter Polymorphisms and the Development of Albuminuria in Type 2 Diabetes: A Case–Control Study: Erratum

**DOI:** 10.1097/01.md.0000479541.52095.5b

**Published:** 2016-01-08

In the article “Association Between Heme Oxygenase-1 Promoter Polymorphisms and the Development of Albuminuria in Type 2 Diabetes: A Case–Control Study”,^1^ which appeared in Volume 94, Issue 43 of *Medicine*, Dr. Kyu Sik Jung's name was incorrectly given as Kyu Sik Chung. The article has since been corrected online.
